# *Dirofilaria immitis* Pulmonary Dirofilariasis, Slovakia

**DOI:** 10.3201/eid2802.211963

**Published:** 2022-02

**Authors:** Martina Miterpáková, Daniela Antolová, Jana Rampalová, Miroslava Undesser, Tomáš Krajčovič, Bronislava Víchová

**Affiliations:** Institute of Parasitology of Slovak Academy of Sciences, Košice, Slovakia (M. Miterpáková, D. Antolová, B. Víchová);; Unilabs Slovakia Ltd., Diagnostic Center of Pathology, Bratislava, Slovakia (J. Rampalová, T. Krajčovič);; Ambulance of Pneumology and Phthisiology, Dunajská Streda, Slovakia (M. Undesser)

**Keywords:** Dirofilaria immitis, nematodes, pulmonary dirofilariasis, Slovakia, parasites

## Abstract

*Dirofilaria immitis* is a parasite related to pulmonary dirofilariasis in humans, its accidental hosts. We detected an autochthonous case of *D. immitis* infection in a woman from Slovakia. The emergence and spread of this parasite in Europe indicates a critical need for proper diagnosis of infection.

*Dirofilaria immitis* is a filarioid nematode that infects numerous mammalian species. Dogs are the main reservoir and various mosquito species (e.g., genera *Culex*, *Anopheles*, *Aedes*, and *Ochlerotatus*) the infection vectors. The parasite is related to the pulmonary form of dirofilariasis, manifested by the formation of coin lesions or nodules in lung parenchyma in humans, an accidental parasite host (*1*).

In Europe, the species *D. repens* causes most reported cases of human dirofilariasis. Just over 30 cases of human *D. immitis* infection have been unambiguously diagnosed, compared with >4,000 from *D. repens* (*2*).

In Slovakia, human autochthonous dirofilariasis has occurred since 2007. Meanwhile, 24 cases have been confirmed, all caused by *D. repens* nematodes (*3*,*4*; M. Miterpáková, unpub. data).

In January 2020, a 66-year-old woman from southwestern Slovakia was admitted to the Ambulance of Pneumology and Phthisiology reporting chest pain, cough, and asphyxia. The patient, who had a long-term history of smoking, had been treated for bronchial asthma and chronic obstructive pulmonary disease since 2012. She had not been abroad for >5 years. Results of hematologic and biochemical examinations were within the physiologic ranges; pulmonary function tests revealed moderate obstructive pulmonary disorder: a decrease of vital capacity to 77% (reference range >80%) and forced expiratory volume during the first second to 63% (reference range >80%). A chest radiograph showed bilaterally hyperlucent lungs with coarse bronchovascular markings; therefore, emphysema was suspected. Subsequent computed tomography confirmed bilateral paraseptal emphysema and numerous nonspecific lesions, ≈5 mm in diameter, in the S2 segment of the right lung and solitary nodules in the S8/9 segment of the left lung. Because of suspected malignancy, the patient was regularly monitored. After 12 months, the cancer markers had elevated. A control computed tomography examination showed a subpleural focal lesion in the S10 segment of the right lung; positron emission tomography/computed tomography confirmed hypermetabolic activity of the lesion. Biopsy was recommended because of a suspected tumor.

In May 2021, a wedge surgical resection of the nodule was performed. Histologic examination of resected tissue revealed a well-circumscribed necrotic nodule containing small irregularly shaped tubular formations affected by massive degenerative changes. The edge of the nodule consisted of nonspecific fibrotic and inflammatory granulations (Figure). The final pathology report suggested the presence of massively degenerated fragments of a nonvital parasite, with *Dirofilaria* spp. suspected.

**Figure:**
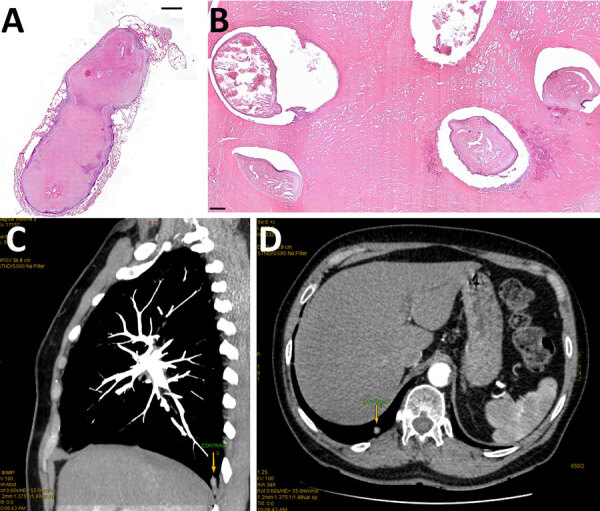
Histologic examination of resected tissue from a 66-year-old woman from southwestern Slovakia. A, B) Cross section showing *Dirofilaria immitis* nematodes embedded in necrotic material obtained from well-defined pulmonary nodule. Hematoxylin and eosin staining; original magnification ×20 for panel A, ×100 for panel. C, D) Chest computed tomography scan showing a subpleural focal lesion in the S10 segment of the right lung (arrows).

We amplified the mitochondrial *cox1* gene fragments of *D. repens* (209 bp) and *D. immitis* (203 bp), in accordance with Rishniw et al. (*5*). The analyzed tissue was positive for *D. immitis* and subsequent sequencing and BLAST analysis of the sequence (GenBank accession no. MZ438680) revealed 100% identity within the region overlapping other homologous *D. immitis* sequences from GenBank (e.g., accession nos. KC985239, NC005305).

Pulmonary dirofilariasis is still very rare in Europe, and many cases are evaluated as imported. Even cases published as autochthonous are still under discussion, and no final decision has been reached. For instance, Pampiglione et al. (*6*) published an analysis of 28 human cases diagnosed in Europe and attributed to *D. immitis* parasites or a species other than *D. repens*. In this analysis the researchers excluded *D. immitis* parasites as a causative agent in all the reviewed cases (*6*). A nonspecific localization of *D. repens* infection in lung tissue was recently reported in several patients from Russia, and 1 case was diagnosed in Slovakia (*3*,*7*). Recent data from several European countries, including Slovakia, indicate dramatic increase of *D. immitis* infections in the canine population (*4*,*8*,*9*), which may cause a rise in human cases in the near future.

Human pulmonary dirofilariasis is characterized by the formation of typical nodules (coin lesions) around immature adult worms located mainly on the lung periphery (*6*). Differential diagnosis of the nodules is important because >20 other pathologic conditions manifests by coin lesions, including tumors, cysts, and inflammatory granulomas. Coin lesions observed in patients with pulmonary dirofilariasis are spherical, not pyramidal as embolic infarct, and generally range from 1 cm to 4.5 cm in diameter (*10*). Few patients with pulmonary dirofilariasis show clinical symptoms. When symptoms are present, they are nonspecific and include thoracic pain, cough, and purulent sputum. These symptoms imitate pneumonitis, and patients are often treated incorrectly with antimicrobial drugs (*1*).

Long-term experience from dirofilariasis-endemic areas confirms that diagnosis is key and still a great challenge in the successful encompassment of human pulmonary dirofilariasis. Given the lack of specific and sensitive serologic tests, the only way for correct presurgical diagnosis appears to be the use of medical imaging. According to the European Society of Dirofilariosis and Angiostrongylosis (*2*), the combination of ultrasound and color Doppler charting, which offers findings of well-defined characteristics of *D. immitis* nodules (e.g., regular oval shape, hypoechoic inner content, no signs of polar vascularity) enable attribution to helminthic origin.
